# Changes in Hepcidin Serum Levels Correlate with Clinical Improvement in Idiopathic Restless Legs Syndrome Patients

**DOI:** 10.3390/jcm9124115

**Published:** 2020-12-20

**Authors:** Hee-Jin Im, Jee Hyun Kim, Chang-Ho Yun, Dong Wook Kim, Jeeyoung Oh

**Affiliations:** 1 Department of Neurology, Dongtan Sacred Heart Hospital, Hallym University College of Medicine, Hwaseong 18450, Korea; coolere@naver.com; 2 Department of Neurology, Ewha Womans University Seoul Hospital, Ewha Womans University School of Medicine, Seoul 07804, Korea; fever26@gmail.com; 3 Department of Neurology, Seoul National University Bundang Hospital, Seongnam 13620, Korea; ych333@gmail.com; 4 Department of Neurology, School of Medicine, Konkuk University Hospital, Konkuk University, Seoul 05030, Korea; drdongwkim@gmail.com

**Keywords:** hepcidin, treatment response, restless legs syndrome, IRLS, quality of life

## Abstract

Background: Restless legs syndrome (RLS) is a common sensory motor neurological disorder that is related to iron–dopamine dysregulation and immune system alteration. We aimed to assess the effects of serum hepcidin, an iron-regulating hormone, in drug-naive RLS patients compared to healthy controls and to evaluate its role in helping to predict clinical improvement after treatment with dopamine agonist. Methods: Nonanemic and drug-naive RLS patients (*n* = 18) and healthy controls (*n* = 15) were enrolled. The serum hepcidin and iron-related values in the serum were measured upon the first visit in both groups and 12 weeks later after dopaminergic treatment in 12 patients. Information about sociodemographic characteristics, sleep-related profiles, mood and anxiety was obtained upon the first visit in all participants as well as after treatment in RLS patients. Results: Serum hepcidin levels exhibited no significant differences between patients with drug-naïve RLS and healthy controls at diagnosis (7.1 ± 2.4 vs. 7.0 ± 3.2 ng/mL, *p* = 0.357). Decreased hepcidin levels were significantly associated with decreased RLS severity (β = 0.002, 95% CI = 0.00−0.00, *p* = 0.005) and improved quality of life (β = 0.002, 95% CI = 0.00−7.01, *p* = 0.044) in a dose-dependent manner after 12 weeks of treatment with a dopamine agonist. This association was independent of age, sex, inflammatory markers, sleep quality, insomnia, daytime sleepiness, depression and anxiety. Conclusions: This study demonstrates the role of hepcidin in evaluating the positive therapeutic response in RLS.

## 1. Introduction

Restless legs syndrome (RLS), recently also called Willis–Ekbom disease, is a neurological sensorimotor disorder characterized by an urge to move the legs during rest that leads to sleep disturbance, especially at the initiation of sleep, and can even impair quality of life during the day [1]. The biological basis of RLS includes genetic variations, dopamine dysregulation and inflammation; however, its pathophysiology still remains inconclusive. Research has reached a consensus about the underlying cause of RLS and has demonstrated that it involves a dysregulation of iron availability in the brain, which is supported by the following considerations. First, RLS is a common comorbid disease in patients with conditions related to consuming or deficiency of iron, including anemia, renal failure, pregnancy, liver disease and absorption diseases, such as irritable bowel syndrome [2]. Second, treatment with oral or intravenous iron is effective even in patients with idiopathic RLS [3,4]. Third, decreased levels of D2 receptor in the putamen were correlated with the severity of RLS and increased levels of tyrosine hydroxylase in the substantia nigra were found in animal and autopsy models of iron insufficiency [5,6]. The diagnosis and disease severity of RLS are still based on the clinical symptoms of RLS, which may lead to an over- or underdiagnosis of RLS [7,8,9]. Low serum ferritin levels are representative of a deficit in body iron stores and are also known to be a strong predictive marker of the severity of symptoms in patients with RLS [10,11].

Hepcidin is the key regulatory hormone of systemic iron homeostasis, initially produced by hepatocytes as an 84-amino acid peptide (pre-prohepcidin). It is processed by a plasma peptidase into a biologically active 25-amino acid form (hepcidin-25) and 60-amino acid prohepcidin, which are excreted through urine [12,13]. Hepcidin-25 binds to iron transporter ferroportin on the surface of erythrocytes and macrophages, which limits iron movement from cells. Consequently, hepcidin decreases iron uptake in the gut. Beyond the involvement of iron metabolism, hepcidin is also closely linked with inflammation and infection. Serum pro-hepcidin and hepcidin-25 have been shown to reflect the pathology of inflammation, such as rheumatoid arthritis and irritable bowel syndrome [14]. To date, only two studies have investigated the hepcidin levels in patients with RLS. One study demonstrated that pro-hepcidin levels were decreased in the cerebrospinal fluid (CSF) during early-onset RLS [15] and the other study demonstrated increased serum hepcidin-25 levels in patients with idiopathic RLS compared to healthy controls [16].

In this study, we aimed to evaluate the association between human hepcidin level in RLS patients compared to healthy controls and therapeutic responses after dopaminergic treatment.

## 2. Methods

### 2.1. Participants

This case-control study was conducted at a single tertiary hospital. Participants who met the inclusion criteria were recruited from May 2017 to February 2018 at the Sleep Clinic in the Department of Neurology of Korea University Anam Hospital (Seoul, Republic of Korea). The inclusion criteria for the patient group were as follows: (1) this was the patient’s first visit due to leg discomfort; (2) the patient was over 19 years old; (3) the patient’s symptoms met the diagnostic criteria for RLS according to the International Restless Legs Syndrome Study Group (IRLSSG) [7]; (4) the participants had neither taken medication for their leg symptoms nor had anemia before; (5) the participants did not have a history or evidence of anemia, renal or liver disease, pregnancy, neurological disorders (e.g., Parkinson’s disease or polyneuropathy), any inflammatory disease or current infection. For the control group, 15 volunteers were recruited who had no sleep complaints, no current or past RLS symptoms and no history of anemia, iron deficiency, renal or liver disease or neurologic or inflammatory disorders. The exclusion criteria were as follows: (1) the participants had undergone dopaminergic treatment or treatment of anemia previously and (2) the participants were taking one or more medications for sleep-related problems. All participants were interviewed using a booklet questionnaire and evaluated by an experienced neurologist (HJ Im) who was trained in sleep medicine to confirm the presence of definite RLS. All participants were examined with a nerve conduction study and electromyography of their legs to assess the presence of peripheral neuropathy. The patients were treated for 12 weeks with 0.125–0.25 mg of pramipexole 1–2 h before bedtime; the control participants were interviewed upon their first visit and the patient group participants were interviewed both upon their first visit and after 12 weeks of treatment. Out of nineteen patients and fifteen controls, 12 patients were included for serial subgroup analysis after excluding seven patients who did not complete the questionnaire or did not undergo blood sampling after 12 weeks of treatment. This study was approved by the institutional review board of Korea University Anam Hospital (no. ED16224). Informed consent was obtained from all individual participants included in the study.

### 2.2. Assessment of the Severity and Impact of RLS on the Quality of Life

The severity of RLS symptoms was assessed by the Korean version of the International Restless Legs Syndrome Study Group rating scale as a continuous value (K-IRLS); a score of > 20 indicated severe RLS symptoms [8,17]. To evaluate the quality of life of RLS patients, a validated Korean version of the Restless Legs Syndrome Quality of Life questionnaire (K-RLSQoL) was adopted; higher scores indicated a better quality of life [1,17].

### 2.3. Blood Workup

Venous blood sampling was performed between 09:00 and 09:30 a.m. after overnight fasting to minimize diurnal variation of serum hepcidin levels for baseline and follow-up after 12 weeks of treatment. Serum chemistry, including liver, renal and thyroid function tests, were performed to differentiate from secondary RLS and C-reactive protein as an inflammatory parameter was assessed. To assess the iron profile, serum hemoglobin, ferritin, iron, transferrin and the total iron-binding capacity (TIBC) level were quantified. Blood samples were taken from patients and controls at the same time upon their first visit.

### 2.4. Assessment of Hepcidin

Serum hepcidin (pre-prohepcidin) was measured using the human hepcidin immunoassay kit (R&D Systems Inc., Minneapolis, MI, USA) following the manufacturer’s instructions. Pre-prohepcidin is an initially synthesized peptide which is processed into hepcidin-25 and prohepcidin in hepatocytes. The samples of both patients and controls were acquired between 09:00 and 09:30 a.m. after overnight fasting and were measured in triplicate using an enzyme-linked immunosorbent assay (ELISA). In the patient group, samples were acquired again in the same manner after 12 weeks of treatment. The concentration of hepcidin was presented as the average of the triplicate samples.

### 2.5. Assessment of Sleep Parameters and Mood Status

Information regarding the sleep—wake schedules on workdays and free days was obtained. The average weekly sleep duration was calculated as a weighted value, based on workdays and free days, as follows: (5× sleep duration on workdays + 2× sleep duration on free days)/7. Sleep quality was assessed using the Pittsburgh Sleep Quality Index and a score of >5 indicated poor sleep quality [18]. Excessive daytime sleepiness was defined as an Epworth Sleepiness Scale score of >10 [19]. Insomnia was evaluated using the Insomnia Severity Index, and a score of >14 suggested moderate-to-severe insomnia [20]. The presence and severity of a depressed mood was evaluated using the Patient Health Questionnaire-9 (PHQ-9); a total score of ≥10 suggested the presence of depression [21]. Anxiety was defined by a Beck’s Anxiety Inventory score of ≥22 [22].

### 2.6. Statistical Analyses

Continuous variables were presented as means with standard deviation and categorical variables as numbers with percentages. The Kolmogorov–Smirnov test was used to test the normality of continuous variables. For group comparisons between the patients and controls, a two-tailed Student’s t-test/Mann–Whitney U test or chi-squared test/Fisher test was used for continuous or categorical variables, respectively. For the patient group analysis, we conducted multiple linear regression analysis; general linear model analysis was used to test the association of hepcidin levels with the improvement of RLS symptoms and quality of life after 12 weeks of treatment. We used the main dependent and independent variables for individual differences with the paired value before and after treatment. Age, sex, C-reactive protein (CRP), depression, anxiety, sleep quality and insomnia were adjusted as continuous variables. The data were analyzed using SPSS version 24.0 (SPSS, Inc., Chicago, IL, USA). A *p*-value of <0.05 indicated a statistical significance.

## 3. Results

### 3.1. Demographic Data and Subjective Sleep Scales

A total of 33 participants enrolled in the study (21 females, 63.6%; mean age: 43.4 ± 13.0 years). The average age and proportion of female RLS patients was comparable with that of controls (47.3 ± 12.1 years vs. 38.8 ± 12.9 years, *p* = 0.061; 66.7% vs. 60.0%, *p* = 0.692, Table 1). The mean IRLS score of patients was 20.1 ± 11.2 and 50.0% of patients reported severe RLS symptoms (IRLS score > 20). The quality of life of RLS patients was poorer than healthy controls (RLSQoL score: 36.6 ± 9.4 years vs. 48.3 ± 2.9, *p* < 0.0001). Overall sleep parameters of patients were impaired compared to controls. The following variables were significantly higher in patients than in controls: poor sleep quality (Pittsburgh Sleep Quality Index (PSQI) score: 10.0 ± 5.2 vs. 5.7 ± 3.4, *p* = 0.009), anxiety (Beck Anxiety Inventory (BAI) score: 11.4 ± 12.7 vs. 3.3 ± 4.1, *p* = 0.011), severe insomnia symptom (Insomnia Severity Index (ISI) score: 21.7 ± 12.7 vs. 4.5 ± 4.0, *p* = 0.002) and the proportion of participants with moderate-to-severe insomnia (ISI score ≥ 15: 44.4% vs. 0.0%, *p* = 0.004). The severity of depressed mood was also higher than controls with borderline significance (PHQ-9 score: 7.2 ± 7.2 vs. 2.9 ± 3.2, *p* = 0.070). Subjective sleep parameters also revealed greater sleep disturbance in RLS patients compared with controls (Table 1): shorter weekly sleep duration (5.4 ± 1.8 h vs. 6.4 ± 0.9 h, *p* = 0.067) and a higher proportion of poor sleep quality (PSQI > 6: 78.9% vs. 46.7%, *p* = 0.064). Except for a few participants taking drugs for antihypertension and thyroid hormones, none of the participants were taking other drugs that could affect RLS symptoms.

### 3.2. Hepcidin Levels

Compared to healthy controls, variables related to iron homeostasis, including the levels of serum hemoglobin, ferritin, iron and transferrin, as well as TIBC, did not differ in patients with drug-naive RLS at diagnosis. Hepcidin level at baseline but did not differ between groups (7.1 ± 2.4 vs. 7.0 ± 3.2 ng/mL, *p* = 0.357; Table 2). Hepcidin level was comparable even between two groups with normal ferritin. The ferritin concentration was <50ng/mL in four RLS patients and five controls; however, no other secondary RLS-related factors, such as abnormal liver, renal or thyroid function, were demonstrated with the blood analysis. Electrophysiological tests including, nerve conduction study and electromyographic study, showed no abnormalities among all participants.

### 3.3. RLS severity is Associated with Hepcidin Levels after Treatment

The mean baseline value of hepcidin levels before treatment of the patients was 7.1 ± 2.4 ng/mL and after 12 weeks of treatment the mean value was 6.3 ± 2.5 ng/mL. The mean individual difference of hepcidin levels before and after treatment was 0.6 ± 3.4 ng/mL. The mean individual decrease of IRLS score after 12 weeks of treatment compared with baseline was 10.3 ± 4.8 and the proportion of dopamine agonist treatment response (more than 50% decrease of IRLS score) was 33.3%.

The multimodel analysis revealed that the improvement of RLS symptoms (a decrease in IRLS scores) after dopaminergic treatment (0.125–1.25 mg of pramipexole for 12 weeks) was positively linearly associated with the decrease in hepcidin levels after treatment (Table 3). The more the symptoms were improved, the lower the hepcidin levels were, independent of inflammation, age, sex, sleep disturbance (e.g., sleep quality, daytime sleepiness, insomnia severity) and mood status (e.g., depression, anxiety), 12 weeks after treatment (β = 0.002, 95% CI 0.00–0.00, *p* = 0.005). The improvement of symptoms and younger age were also associated with borderline significance (β = 0.002, 95% CI 0.00–0.00, *p* = 0.005). The linear relationship between IRLS scores and hepcidin levels after treatment was still significant in patients with normal ferritin, i.e., > 50 ng/mL (β = −0.15, 95% CI −0.11–0.31, *p* = 0.057). On the other hand, the difference in serum ferritin levels before and after treatment was not significantly correlated with the difference in hepcidin levels and IRLS scores in the group of RLS patients.

### 3.4. Quality of Life is Associated with Hepcidin Levels After Treatment

**Figure 1:**
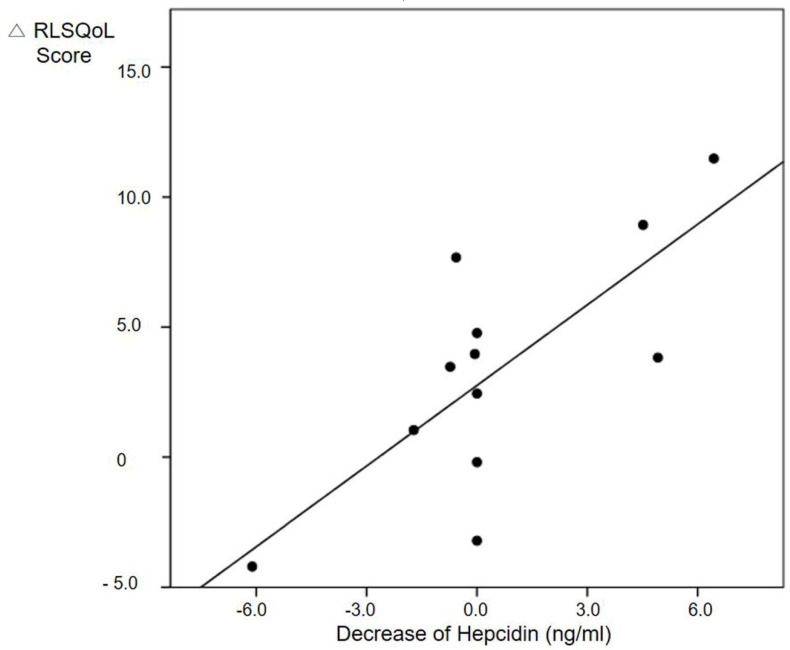
Linear association between improvement of quality of life and decrease of hepcidin in RLS patients 12 weeks after treatment. Abbreviations: RLS, restless legs syndrome; RLSQoL, Restless Legs Syndrome Quality of Life score.

The decrease in hepcidin levels was significantly associated with the improvement of quality of life in RLS patients after 12 weeks of treatment (Figure 1). A linear association was observed between hepcidin levels and RLSQoL scores after adjusting confounding factors such as age, sex, difference of CRP, depression, anxiety and insomnia scores after treatment (β = 0.002, 95% CI 0.00–7.01, *p* = 0.044). The linear relationship between quality of life and hepcidin levels after treatment was still significant in patients with high normal ferritin, i.e., >50 ng/mL (β = 0.002, 95% CI 0.00–0.00, *p* = 0.043).

## 4. Discussion

In this study, we aimed to investigate the roles of serum hepcidin measured as pre-prohepcidin as a biomarker in helping in the prediction of treatment response in RLS. The main findings of the present study were as follows: (1) hepcidin levels in RLS patients and healthy controls did not differ at diagnosis, even in patients with normal ferritin levels. (2) The extent of decrease of serum hepcidin levels was a strong predictor of symptom improvement in a dose-dependent manner after 12 weeks of dopaminergic treatment, independent of age, sex, CRP, sleep quality, insomnia, daytime sleepiness, depression and anxiety. (3) The extent of decrease of serum hepcidin levels was also linearly associated with an improvement of quality of life in RLS patients after 12 weeks of dopaminergic treatment after adjusting for age, sex, CRP, depression, anxiety and insomnia severity.

Hepcidin, an important regulator of iron metabolism, interacts with ferroportin to induce cellular internalization, which leads to a reduction in iron in the periphery through signaling with erythrocytes and macrophages [23]. The synthesis of hepcidin is greatly stimulated by inflammation or by iron overload, which results in hyperstimulation during inflammatory states and thus in iron-restricted erythropoiesis [24]. Increased hepcidin was also found in general inflammatory disorders such as rheumatoid arthritis and inflammatory bowel disease in humans [14,25,26,27]. The presumptive connection effected by hepcidin between iron and inflammation in the pathogenesis of neural diseases is also apparent in the central nervous system(CNS). For example, inflammatory substances such as lipopolysaccharide and endotoxin induce hepcidin expression in the substantia nigra of the brain and lead to iron retention in neurons in an inflammatory-related Parkinson’s disease rodent model [27,28]. Current research on the pathophysiology of RLS has demonstrated dopamine dysregulation and altered iron homeostasis (including iron depletion) as causative factors. Furthermore, systemic inflammation may play a role in RLS in a reciprocal manner, which is supported by high serum neutrophil-to-lymphocyte ratio [29].

To date, several studies have investigated hepcidin in RLS. One study evaluated patients with early-onset RLS (in which symptoms were present before the age of 45); compared to controls, these patients exhibited lower pro-hepcidin and ferritin levels in the CSF and conversely higher pro-hepcidin and ferritin levels in neuromelanin cells in the brain (substantia nigra and putamen) [15]. The patients were divided into groups based on the age of symptom onset, not according to iron status or other pathological basis. These patients were taking dopaminergic agents in various doses; thus, the possibility of assessing the diagnostic value of hepcidin before treatment of RLS was limited, as the potentially complex interactions between dopamine and iron homeostasis must be considered. A recent large study has demonstrated a significant association between primary RLS and increased serum hepcidin-25 levels, but not ferritin levels. It also showed a U-shape relationship between hepcidin-25 and RLS severity [16]. These results could suggest that serum hepcidin level can be associated with RLS severity; however, the study did not use serial analysis for each individual and did not consider other sleep-related factors that could affect the severity of RLS.

This current study has many strengths. First, this is the first study to evaluate the association between serum hepcidin (pre-prohepcidin) and drug-naive RLS patients regardless of serum ferritin levels. Second, we demonstrated a clear dose-dependent association between hepcidin and therapeutic response with regard to the improvement of symptom severity and quality of life in RLS patients after 12 weeks of controlled treatment. Third, the complex relationships of various potential factors affecting the severity of RLS symptoms and quality of life (i.e., age, sex, poor sleep quality, insomnia, daytime sleepiness, depression and anxiety) were taken into consideration. Fourth, to the best of our knowledge, this is the first study to include inflammation markers as a covariate in the analysis of hepcidin level changes in RLS.

Nevertheless, this current study has several limitations as well. First, this study was conducted in a single tertiary sleep center and comprised a relatively small sample size; further studies in a multicenter setting with a higher sample number would verify the findings. Second, from a critical point of view, our study showed notably higher hepcidin levels in the RLS group, which is similar to a recent report which indicated a positive relationship between serum hepcidin-25 and primary RLS based on larger sample sizes [16], but did not reveal significant differences in hepcidin levels compared to the control group. This may be because we utilized pre-prohepcidin as a precursor to hepcidin-25 or because of the small sample size. However, the disease manifestations of RLS depend on life span and age at onset and hepcidin levels and iron metabolism also change with age in the general population, especially in women [15,30,31]. A higher age (albeit with borderline significance) in the patient group than in the control group may have played a role in the absence of differences in hepcidin levels between groups. Third, the findings of this study were based on 12 weeks of treatment and there was no long-term investigation regarding the association between therapeutic responses and hepcidin levels; additional longitudinal research is needed. Unlike our results, a recently published report showed no difference in hepcidin levels after 13 months of treatment in 37 RLS patients. However, this finding was not based on each individual difference, was inhomogeneous of type and dose of treatment drugs and, in addition, some patients had a history of previous augmentation (albeit not at the analysis) [32]. Fourth, the analysis of the current study could have used a selection-biased result with regard to the patients who were treatment-effective at 12 weeks of during the follow-up period. Fifth, one of the main complications of dopaminergic treatment of RLS, augmentation of symptoms, must be considered in the therapeutic response of long-term treatment. Sixth, the primary outcomes of the study (the severity of RLS symptoms (IRLS score) and effects on quality of life (RLSQoL score)) were based on a questionnaire; objective measurements of therapeutic responses, such as polysomnography parameters, are needed for further analysis.

The causal relationship and regulatory mechanisms between dopamine, iron and inflammation in the brain and periphery, as well as the interaction between hepcidin, prohepcidin and hepcidin-25, remain to be elucidated. It is still unclear how pre-prohepcidin effects transcription or processing into serum hepcidin-25 and pro-hepcidin, in serum as well as in the CNS, and further investigation is needed. Hepatocellular pre-prohepcidin expression responds to increases in serum or tissue iron triggers. Under the conditions of an erythropoietic drive, hepcidin expression is suppressed and leads to iron mobilization for erythropoiesis [33]. Likewise, increasing availability of dopamine in the CNS through dopamine agonist may increase iron mobilization as a cofactor of dopamine synthesis and eventually lead to a decrease of pre-prohepcidin levels (this may be parallel with active 25-hepcidin). A persuasive association between dopamine agonist and increased iron homeostasis and metabolism was reported in a recent in vitro study [34]. The expansion of intracellular iron upon transcription of iron export protein ferroportin as a result of dopamine treatment was significantly increased, suggesting that dopamine directly affects cellular iron homeostasis by increasing iron incorporation into macrophages. The decrease in serum hepcidin levels after dopaminergic treatment suggests that dopamine supplement can improve the bio-availability of iron, thus alleviating iron deficiency in the CNS and leading to the clinical improvement of disease severity and quality of life of patients.

## 5. Conclusions

Hepcidin is a key iron-regulating hormone that can serve as an important mediator of immune and iron metabolism, both of which precede the pathophysiology of RLS. This study demonstrates the potential role of hepcidin in helping to predict positive therapeutic response in RLS patients.

## Figures and Tables

**Table 1:** Demographic and clinical characteristics of restless legs syndrome (RLS) patients and the control group.

Characteristics	Patient (*N* = 18)	Control (*N* = 15)	*p*-Value
Age (y)	47.3 ± 12.1	38.8 ± 12.9	0.061
Female sex	12 (66.7)	9 (60.0)	0.692
BMI (kg/m^2^)	22.3 ± 3.0	21.3 ± 5.5	0.762
Weekly sleep duration (hour)	5.4 ± 1.8	6.4 ± 0.9	0.067
Sleep efficiency (%)	78.6 ± 22.3	90.3 ± 13.9	0.229
Sleep latency (min)	51.4 ± 46.4	30.1 ± 30.8	0.117
PSQI score	10.0 ± 5.2	5.7 ± 3.4	0.009 *
Poor sleep quality (PSQI > 5)	14 (77.8)	7 (46.7)	0.064
Epworth Sleepiness Scale	6.8 ± 5.0	5.0 ± 3.0	0.220
Excessive daytime sleepiness (ESS > 11)	4 (22.2)	0 (0)	0.108
Insomnia Severity Index	21.7 ± 40.8	4.5 ± 4.0	0.002 *
Moderate to severe insomnia (ISI ≥ 15)	8 (44.4)	0 (0)	0.004 *
Beck Anxiety Inventory score	11.4 ± 12.7	3.3 ± 4.1	0.011 *
Moderate anxiety (BAI ≥ 22)	3 (16.7)	0 (0)	0.233
PHQ-9 Depression Scale score	7.2 ± 7.2	2.9 ± 3.2	0.070
Depressive mood (PHQ-9 ≥10)	4 (22.2)	1 (6.3)	0.346

The data are presented as means ± the standard deviation or as numbers (%). * *p* < 0.05, based on Student’s t-test/Mann–Whitney U test or chi-squared test/Fisher’s test. BMI, body mass index; PSQI, Pittsburgh Sleep Quality Index; ESS, Epworth Sleepiness Scale; ISI, Insomnia Severity Index; BAI, Beck Anxiety Inventory; PHQ-9, Patient Health Questionnaire-9.

**Table 2:** The difference in iron profiles including hepcidin between RLS patients and the control group.

Characteristics	Patient (*N* = 18)	Control (*N* = 15)	*p*-Value
Ferritin level (ng/mL)	85.9 ± 61.7	112.4 ± 149.6	0.901 ^α^
Hepcidin level (ng/mL)	7.1 ± 2.4	7.0 ± 3.2	0.357 ^α^
Hemoglobin (g/dL)	13.2 ± 1.7	14.2 ± 1.5	0.120
TIBC (μg/dL)	315.4 ± 27.2	327.0 ± 41.4	0.344
Iron (μg/dL)	101.4 ± 44.1	82.0 ± 29.1	0.154
Transferrin (mg/dL)	253.5 ± 20.4	258.3 ± 30.3	0.602

The data are presented as means ± the standard deviation. ^α^. TIBC, total iron binding capacity.

**Table 3:** Predictors for relief of RLS symptoms three month after treatment (△IRLS score).

Variables	Model 1		Model 2		Model 3	
	β (95% CI)	*p* Value	β (95% CI)	*p* Value	β (95% CI)	*p* Value
Decrease of hepcidin level (ng/mL)	0.001 (0.00, 3.96)	0.042 *	0.001 (0.00, 0.00)	0.025 *	0.002 (0.00, 0.00)	0.005 *
Difference of CRP level	0.230 (−0.78, 1.24)	0.617	0.041 (−1.01, 1.10)	0.928	−0.705 (−1.92, 0.51)	0.130
Age (years)			−0.172 (−0.49, 0.15)	0.241	−0.148 (−0.11, 0.31)	0.057
Male sex			2.394 (−3.45,8.24)	0.365	0.690 (−3.30, 4.68)	0.534
Sleep quality (PSQI score)					−0.379 (−1.37, 0.61)	0.241
Insomnia severity (ISI score)					−1.192 (−1.92, 0.46)	0.020 *
Daytime sleepiness (ESS score)					−1.328 (−2.23, −0.43	0.014 *
Depression severity (PHQ-9 score)					4.872 (2.58, 7.17)	0.012 *
Anxiety severity (BAI score)					−1.328 (−2.23, −0.43)	0.024 *

Data presented as correlation coefficient (β, 95% CI) from generalized linear model analysis; * *p* < 0.05. CI, confidence interval; IRLS, International Restless Legs Syndrome Study Group rating scale; PSQI, Pittsburgh Sleep Quality Index; ISI, Insomnia Severity Index; ESS, Epworth Sleepiness Scale; PHQ-9, Patient Health Questionnaire-9; BAI, Beck Anxiety Inventory.
